# Depression and quality of life among pregnant women in first and third trimesters in Abeokuta: A comparative study

**DOI:** 10.4102/sajpsychiatry.v28i0.1779

**Published:** 2022-03-31

**Authors:** Adetoun O. Soyemi, Oladipo A. Sowunmi, Sunday M. Amosu, Emmanuel O. Babalola

**Affiliations:** 1 Southwest Yorkshire NHS Trust, Wakefield, United Kingdom; 2 Neuropsychiatric Hospital Aro, Abeokuta, Ogun State, Nigeria; 3 Hywel Dda NHS Trust, Carmarthen, Wales, United Kingdom

**Keywords:** depression, quality of life, pregnant women, mental health, psychological disorder, trimesters

## Abstract

**Background:**

Pregnancy is a dynamic time during which a woman’s emotional state may undergo extensive change. There have been conflicting views about the magnitude of emotional turmoil that occurs during pregnancy. Some investigators suggest that pregnancy is a time of particularly good psychological adjustment; others have reported high levels of psychological challenge.

**Aim:**

Our study aimed to compare the prevalence and correlates of depression in the first and third trimesters of pregnancy and to determine the relationship between quality of life and depressive disorder.

**Setting:**

The antenatal clinic of the State Hospital, Ijaiye.

**Method:**

A descriptive, comparative study of depressive disorder and the quality of life between first- and third-trimester pregnant women (confirmed through a pregnancy test and an abdominopelvic ultrasound).

**Result:**

For each trimester, 285 participants were recruited. The prevalence of depression among the pregnant women who participated in the study was 7.2%. In the first trimester of pregnancy, the prevalence of depression was 30 (10.5%), while it was 11 (3.9%) in the third trimester of pregnancy. Collectively, the relationship between depression and QoL was significant in the overall domain, satisfaction with general health domain (*t* = 2.27; *p* = 0.03), psychological domain (*t* = 2.74; *p* = 0.010, and environmental domain (*t* = 4.57; *p* ≤ 0.01).

**Conclusion:**

Our study also highlights the need to pay closer attention to the psychological well-being and quality of life of all pregnant women and not just on their physical health and the baby’s well-being.

## Introduction

Pregnancy is a dynamic time during which a woman’s emotional state may undergo extensive change.^1^ There have been conflicting views about the magnitude of emotional disturbances that occur during pregnancy.^2^ Some investigators suggest that pregnancy is a time of particularly good psychological adjustment; others have reported high levels of psychiatric disturbance.^1,2^ According to the American College of Obstetricians and Gynaecologists, the perinatal period is the time when the risk of psychological disorders such as depression and anxiety disorders in a pregnant woman may increase by several folds.^3^

Depression is a mood disorder that is characterised by a prolonged sadness and marked loss of interest in daily activities as core symptoms lasting for at least two weeks or more. Other symptoms are feeling inadequate and worthless, feeling irritable and resentful, insomnia, appetite changes, decreased energy, lack of concentration and poor memory and thoughts of committing suicide or abortion.^4^ Depression affects about 20% of women during their lifetime, with pregnancy being a period of high vulnerability. Depression during pregnancy is not only the strongest risk factor for post-natal depression but also leads to adverse obstetric outcomes.^5^

During the first trimester, unwanted pregnancy and poor marital relationship commonly lead to emotional distress while fear of childbirth and dysfunctional coping style is associated with disturbance during late pregnancy.^6,7,8^ Other associated correlates of emotional distress during pregnancy include twin pregnancy, single status and first pregnancy, history of abortion, low socioeconomic status and low education.^6,9^ Several risk factors predispose women to depression during pregnancy. Some of them are poor antenatal care, poor nutrition, stressful life events like economic deprivation, gender-based violence and polygamy, history of psychiatric disorders, previous puerperal complications, events during pregnancy like previous abortions and modes of previous delivery like past instrumental or operative delivery. Other factors include age, marital status, gravidity, whether the pregnancy was planned or not, a previous history of stillbirth, previous history of prolonged labour and level of social support.^5,10,11^ Thus, assessment of depression during pregnancy is essential for detecting pregnant women in need of intervention to safeguard the well-being of the mother and baby.^5^ Early detection of depression during pregnancy is critical because depression can adversely affect birth outcomes and neonatal health and, if left untreated, can persist after birth. Untreated postpartum depression can impair mother–infant attachments and have cognitive, emotional and behavioural consequences for children.^12^

Quality of life (QoL) is a term describing an individual’s physical, social and emotional well-being and his or her ability to function in carrying out the tasks of living.^13^ The QoL concept is generally described as multidimensional, comprising an individual’s perceived physical, psychosocial and emotional functioning. Quality of life is different from wealth and material standard, and QoL goes beyond material wealth by including also immaterial and collective components such as freedom, equity, social capital, self-fulfilment and happiness.^14^

The perinatal mental health of women living in low- and lower middle-income countries has only recently become the subject of research, in part because the greater priority has been assigned to preventing pregnancy-related deaths. Moreover, some have argued that in resource-constrained countries, women are protected from experiencing perinatal mental problems through the influence of social and traditional cultural practices during pregnancy and in the postpartum period.^10^ Whether this is true remains a subject of debate.

Psychiatric disorders during pregnancy and the postpartum period include conditions of various severity and aetiology most common of these disorders is depression, which is associated with pregnancy-related deaths by suicide and with developmental delays in children.^15^ The prevalence among pregnant women has been reported to be on the average above 10% with others reporting as high as 20%.^10,16^ Various studies that have been carried out in Nigeria focus on the prevalence of depression in the third trimester and the postnatal period^10,16^ with little or no research carried out in the first trimester. Therefore, the thrust of this study is to compare the prevalence and correlates of depression in the first and third trimesters of pregnancy and to determine the relationship between quality of life and depressive disorder.

## Material and method

The study was conducted in Abeokuta, at the antenatal unit of State Hospital Ijaiye, Abeokuta, Ogun State, Nigeria between March and August 2019. A descriptive, comparative study of depressive disorder and the quality of life among first- and third-trimester pregnant women (confirmed through a pregnancy test and an abdominopelvic ultrasound) attending the antenatal clinic of the state attending the antenatal clinic of the state hospital. A total of 285 participants were recruited for each trimester from the antenatal clinic of the State Hospital Ijaiye, Abeokuta, Ogun State, Nigeria. However, women receiving treatment for a known psychiatric disorder and/or a major medical condition such as hypertension, diabetes and chronic illnesses were excluded from the study. Systematic random sampling technique was used in the study. A sample frame was generated from the day’s clinic register of pregnant women. There are two clinic days weekly, with each clinic day catering for about 20 pregnant women, thus making a total number of 40 pregnant women per week. Furthermore, because of the possible logistic problem, a duration of 24 weeks was envisaged for the duration of data collection; this covered for unforeseen problems. Therefore, the total number of possible pregnant women was (40 × 24) 960.

However, as the total sample size in this study is 285 from each trimester, a sample interval of four (4) (960/285 = 3.4, which is approximately 4) was used. Data were analysed using the Statistical Package for Social Sciences (SPSS) version 20. The World Health Organization Quality of Life Brief (WHOQoL-BREF) produced a quality-of-life profile for each participant. In computing the WHOQoL-Bref scores, analysis was done as reported by Skevington and his other coworkers.^17^

The socio-demographic characteristics of respondents were presented using descriptive statistics frequencies and percentages. The means and standard deviations were calculated for continuous variables. Relationships between variables, for example, socio-demographic variables, obstetric/gynaecological variables versus depression disorder were determined using chi-square for categorical variables and *t*-test for comparison of means. The prevalence of definitive psychiatric morbidity and the different diagnostic entities from the M.I.N.I plus was presented categorically, using frequency distribution tables and percentages. The associations between the domains of QOL and the different socio-demographic characteristics were analysed using either an independent *t*-test or analysis of variance (ANOVA). The associations between the domains of QOL and definitive psychiatric morbidity and the different diagnostic entities derived from the M.I.N.I plus was tested using the independent *t*-test and ANOVA. The level of significance was set at <0.05.

Ethical approval was obtained from the Research and Ethical Committee of the Neuropsychiatry Hospital, Aro, Abeokuta Ogun State, and permission was also obtained from the Ogun State Hospital management board, and the management of the State Hospital, Ijaiye where the study was conducted. The purpose of the study was explained to the clients and consent obtained before administering the questionnaires.

### Ethical considerations

Ethical approval was obtained from the Research and Ethical Committee of the Neuropsychiatry Hospital (PR014/17), Aro, Abeokuta Ogun State and permission was also obtained from the Ogun State Hospital management board, and the management of the State Hospital, Ijaiye, where the study was conducted. The purpose of the study was explained to the clients and consent obtained before administering the questionnaires.

## Results

Table 1 shows the socio-demographic characteristics of respondents. The age of the respondents ranged from 18 to 40 years with a mean of 27.26 ± (4.76). Other details can be found in Table 1. Table 2 shows the obstetrics/gynaecological characteristics of respondents. Collectively, 517 (90.7%) had a planned pregnancy, while 53 (9.3%) had an unplanned pregnancy. Further details are depicted in Table 2. The collective mean score of the domains of QoL is overall QoL 3.99±0.88, general health 3.84±0.95, physical domain 57.09±8.03, psychological domain 65.64±8.90, social relationship domain 72.40±11.94 and environmental domain 70.89±9.84. Table 3 illustrates other details. Table 4 demonstrates the relationship between depression and the socio-demographic characteristics of the respondents. The association between age and depression was statistically significant (*χ*^2^ = 10.76, df = 2, *p* = 0.01). Other sociodemographic details can be seen in Table 4. Table 5 depicts the relationship between depression and clinical/gynaecological variables. Abortion was observed to be a significant variable. Other clinical/gynaecological details can be found in Table 5.

**TABLE 1:** Sociodemographic characteristics.

Variable	First trimester		Third trimester		Both trimester	
	*n*	%	*n*	%	*n*	%
**Age (years)**						
18–25	129	45.2	108	37.9	237	41.5
26–35	134	47.1	157	55.1	291	51.1
> 35	22	7.7	20	7.0	42	7.4
**Marital status**						
Single	73	25.6	4	1.4	77	13.5
Married	212	74.4	281	98.6	493	86.5
**Educational level**						
Primary/secondary education	233	81.8	228	80.9	461	80.9
Tertiary education	52	18.2	57	19.1	109	19.1
**Tribe**						
Yoruba	247	86.7	237	83.2	484	84.9
Others	38	13.3	48	16.8	86	15.1
**Occupation**						
Profession/Civil servant	90	31.6	63	22.1	153	26.8
Artisan/Trader	139	48.8	163	57.2	302	53.0
Student/unemployed	56	19.6	59	20.7	115	20.2
**Family type**						
Monogamous	209	73.3	247	86.7	456	80.0
Polygamous	76	26.7	38	13.3	114	20.0
**Social support**						
Low	23	8.2	41	14.3	64	11.3
Average	257	90.0	218	75.4	475	83.3
High	5	1.8	31	10.3	31	5.4
**Gender-based violence**						
None	266	93.4	269	94.4	535	93.9
Present	19	6.6	16	5.6	35	6.1
**Childhood experience**						
Pleasant	258	90.5	269	94.0	527	92.5
Unpleasant	27	9.5	16	5.6	43	7.5

**TABLE 2:** Obstetrics and gynaecological variable in pregnant women.

Variable	First trimester		Third trimester		Both trimester	
	*n*	%	*n*	%	*n*	%
**Planned pregnancy**						
Yes	256	89.8	261	91.6	517	90.7
No	29	10.2	24	8.4	53	9.3
**Parity**						
Nulliparous	67	23.5	161	56.5	228	40.0
Multipara	218	76.5	124	43.5	342	60.0
**Abortion**						
None	260	91.2	252	88.4	512	89.8
One/More	25	8.8	33	11.6	58	10.2
**Mode of delivery**						
Normal	17	7.8	102	82.3	119	34.8
Assisted	201	92.2	22	17.7	223	65.2

**TABLE 3:** Quality of life scores.

Domain	Both trimester mean (s.d.)	First trimester mean (s.d.)	Third trimester mean (s.d.)
Overall QoL	3.99±0.88	4.04±0.93	3.95±0.70
General health QoL	3.84±0.95	3.44±1.01	4.24± 0.77
Physical domain QoL	57.09±8.03	56.13±7.50	58.06±8.44
Psychological QoL	65.64±8.90	66.07±8.68	65.22±9.10
Social domain QoL	72.40±11.94	71.58±12.8	73.22±10.97
Environmental QoL	70.89±9.84	69.44±10.31	72.34±9.13

**TABLE 4:** Relationship between depression and socio-demographic characteristics of respondent.

Variable	First trimester depression						Third trimester depression						Both trimester depression					
	Yes		No		*χ* ^2^	*P*	Yes		No		*χ* ^2^	*P*	Yes		No		*χ* ^2^	*P*
	*n*	%	*n*	%			*n*	%	*n*	%			*n*	%	*n*	%		
**Age**					**9.07**	**0.01**					1.83	0.40					10.76	**0.00**
18–25	21	16.4	107	83.6	-	-	6	5.6	102	94.4	-	-	27	11.4	209	88.6	-	-
26–35	7	5.2	128	94.8	-	-	5	3.2	152	96.8	-	-	12	4.1	280	95.9	-	-
>35	2	9.1	20	90.9	-	-	0	0.0	20	100.0	-	-	2	4.8	40	95.2	-	-
**Tribe**					0.00	0.10					0.66	0.40						
Yoruba	26	10.5	221	89.5	-	-	8	3.4	229	96.6	-	-	34	7.0	450	93.0	0.14	0.71
Others	4	10.5	34	89.5	-	-	3	6.3	45	93.8	-	-	7	8.1	79	91.9	-	-
**Marital status**					5.53	**0.02**					0.15	0.15					16.10	**0.00**
Single	13	17.8	60	82.2	-	-	1	25.0	3	75.0	-	-	14	18.2	63	81.1	-	-
Married	17	8.0	95	92.0	-	-	10	3.6	271	96.4	-	-	27	5.5	466	94.5	-	-
**Educational level**					0.32	0.32					0.05	**0.03**						
Primary/secondary	27	11.6	206	88.4	-	-	6	2.6	222	97.4	-	-	33	7.2	428	92.8	0.00	0.95
Tertiary	3	8.8	49	94.2	-	-	5	8.8	52	91.2	-	-	8	7.3	101	92.7	-	-
**Occupation**					4.02	0.13					0.64	0.73					2.63	0.27
Profession/civil servant	5	5.7	82	94.3	-	-	3	4.8	60	95.2	**-**	**-**	8	5.3	142	94.7	-	-
Artisan/trader	16	11.3	126	88.7	-	-	5	3.1	158	96.9	**-**	**-**	21	6.9	284	93.1	-	-
Student/unemployed	9	16.1	47	94.2	-	-	3	5.1	56	94.9	**-**	**-**	12	10.4	103	89.6	-	-
**Family type**					6.85	**0.01**					1.00	1.00					2.05	0.15
Monogamous	25	9.3	243	90.7	-	-	10	4.0	243	96.0	-	-	35	6.7	486	93.3	-	-
Polygamous	5	29.4	12	70.6	-	-	1	3.1	31	96.9	-	-	6	12.2	43	87.8	-	-
**Social support**					0.28	0.28					0.20	0.20					1.51	0.22
Low	4	17.4	19	82.6	-	-	3	7.3	38	92.7	-	-	7	10.9	57	89.1	-	-
Average/high	26	9.9	236	90.1	-	-	8	3.3	236	96.7	-	-	34	6.7	472	93.3	-	-
**Gender-based violence**					0.70	0.70					0.48	0.48					1.00	0.10
None	1	5.3	18	94.7	-	-	1	6.3	15	93.8	-	-	2	5.7	33	94.3	-	-
Present	29	10.9	237	89.1	-	-	10	3.7	259	96.3	-	-	39	7.3	496	92.7	-	-
**Childhood experience**					2.03	0.13					0.48	1.00					3.32	0.07
Pleasant	25	9.7	234	90.3	-	-	11	4.0	267	96.0	-	-	36	6.7	501	93.3	-	-
Unpleasant	5	19.2	21	80.8	-	-	0	0.0	7	100	-	-	5	15.2	28	84.8	-	-

**TABLE 5:** Relationship between depression and obstetrics/gynaecological variables.

Variables	First trimester depression						Third trimester depression						Both trimester depression					
	Yes		No		*χ* ^2^	*P*	Yes		No		*χ* ^2^	*P*	Yes		No		*χ* ^2^	*P*
	*n*	%	*n*	%			*n*	%	*n*	%			*n*	%	*n*	%		
**Planned pregnancy**					0.75	0.75					0.23	0.23					0.79	0.54
Yes	28	10.9	228	89.1	-	-	9	3.4	252	96.6	-	-	37	7.2	480	92.8	-	-
No	2	6.9	27	93.1	-	-	2	8.3	22	91.7	-	-	4	7.5	49	92.3	-	-
**Parity**					10.00	**0.01**					0.22	0.22					0.28	0.60
1st pregnancy	14	20.9	53	79.1	-	-	4	2.5	157	97.5	-	-	18	7.9	210	92.1	-	-
2nd/>	16	7.3	202	92.7	-	-	7	3.6	117	94.4	-	-	23	6.7	319	93.3	-	-
**Abortion**					109.96	**0.00**					0.49	0.37					72.04	**0.00**
Yes	18	72.0	7	28.0	-	-	2	6.1	31	93.9	-	-	20	34.5	38	65.5	-	-
No	12	4.6	248	95.4	-	-	9	3.6	243	96.4	-	-	21	4.1	491	95.9	-	-
**Mode of delivery**					8.32	**0.00**					0.22	0.22					0.00	0.92
Normal	12	21.1	45	78.9	-	-	4	2.5	157	97.4	-	-	16	7.3	202	92.1	-	-
C/S	18	7.9	210	92.1	-	-	7	5.6	117	94.4	-	-	25	7.1	327	92.9	-	-

The prevalence of depression among the pregnant women who participated in the study was 7.2%. In the first trimester of pregnancy, the prevalence of depression was 30 (10.5%), while it was 11 (3.9%) in the third trimester of pregnancy. Table 6 shows the frequency of depressive symptoms among respondents. The following variables were entered into a logistic regression model age, marital status, number of abortions and childhood experience. In the overall population, women with a history of an abortion or miscarriage were independent predictors of depression and were 12 times more likely to be depressed Please expand compared with the other group (OR = 12.35, CI = 5.87–25.98, *p* = 0.01). Thus, having experienced miscarriage was a significant predictor of depression. Similarly, in the first trimester, having experienced miscarriage was an independent predictor of depression and were five times more likely to be depressed compared with the other group (OR = 5.48, CI = 15.46–178.20, *p* = 0.00). However, none of the variables accessed were an independent predictor of depression in the third trimester. Tables 7–9 show the relationship between sociodemographic variables and QoL in both trimesters, first trimester and the third trimester. Age, marital status and family type were observed to be significantly related to QoL. Other details can be found in Table 7. Table 8 shows the relationship between QoL and obstetrics and gynaecological variables. Table 9 shows the relationship between QoL and depressive illness. Collectively, the relationship between depression and QoL was significant in the overall domain, satisfaction with general health domain, psychological domain and environmental domain. Further details can be found in Table 9.

**TABLE 6:** Table showing the frequency of the items of depressive illness.

Variable	Both		First-trimester		Third-trimester	
	*n*	%	*n*	%	*n*	%
Anhedonia	24	58.5	15	50.0	9	81.8
Sleep changes	20	48.8	10	33.3	10	90.9
Appetite change	26	63.4	10	33.3	7	63.6
Psychomotor slowness	27	65.9	17	56.67	10	90.9
Tiredness	17	41.4	8	26.7	9	81.8
Guilt feelings	8	19.5	6	20.0	2	18.2
Concentration changes	4	9.8	1	3.3	3	27.3
Suicidal ideas	0	0.0	0	0.0	0	0.0

**TABLE 7:** Relationship between quality of life sociodemographic characteristics (both trimester).

Variables	QOL-QV Mean (s.d.)	QOL-GH Mean (s.d.)	QOL-PHY Mean (s.d.)	QOL-PSY Mean (s.d.)	QOL-SOC Mean (s.d.)	QOL-ENV Mean (s.d.)
**Age**						
18–25	3.97±0.70	3.82±0.91	55.82±7.89	65.78±9.17	69.92±13.25	68.80±10.63
26–35	3.99±0.83	3.88±0.99	57.69±7.95	64.97±8.91	74.00±10.62	72.32±9.03
> 35	4.19±0.71	3.95±1.13	69.54±5.86	69.54±5.86	75.207±10.49	72.59±8.62
Statistics	*F* = 1.39, *p* = 0.25	*F* = 0.33 *p* = 0.72	*F* = 6.92, *p* = **0.00**	*F* = 4.98, *p* = **0.01**	*F* = 9.14, *p* = **0.00**	*F* = 1.44, *p* = 0.24
**Tribe**						
Yoruba	4.02±0.81	3.88±0.99	57.10±8.15	65.78±8.99	73.35±11.98	70.97±9.17
Others	3.99±0.81	3.83±0.98	57.06±7.41	64.97±8.35	72.23±11.94	70.97±9.96
Statistic	*t* = 0.35, *p* = 0.72	*t* = 0.46, *p* = 0.65	*t* = 0.04, *p* = 0.97	*t* = 0.87, *p* = 0.39	*t* = 0.81, *p* = 0.42	*t* = 0.08, *p* = 0.93
**Marital status**						
Single	3.82±0.97	3.53±0.95	54.79±6.93	65.42±10.00	70.35±13.89	65.10± 13.17
Married	4.02±0.78	3.89±0.98	57.45±8.14	65.68±8.72	72.72±11.59	71.79±8.90
Statistic	*t* = 1.76, *p* = 0.08	*t* = 2.96, *p* = **0.00**	*t* = 2.72, *p* = **0.01**	*t* = 0.23, *p* = 0.82	*t* = 1.42, *p* = 0.16	*t* = 4.21, *p* = **0.00**
**Educational level**						
Primary/secondary	3.96±0.86	3.80±1.10	56.83±8.09	65.60±9.17	72.12±12.30	70.81±10.03)
Tertiary	4.00± 0.80	3.85±0.96	58.20±7.73	65.65±8.84	73.55±10.26	71.20±9.01)
Statistic	*t* = 0.32, *p* = 0.75	*t* = 0.48, *p* = 0.62	*t* = 1.60, *p* = 0.11	*t* = 0.06, *p* = 0.95	*t* = 1.25, *p* = 0.21	*t* = 0.37, *p* = 0.71
**Occupation**						
Professional/civil servant	3.95±0.87	3.78±1.07	57.86±7.81	66.47±9.60	73.33±10.97	71.89±8.74)
Artisan/trader	4.03±0.76	3.88±0.95	57.03±8.34	65.49±8.24	72.84±11.87	70.86±9.48)
Student/unemployed	3.96±0.83	3.81±0.95	56.28±7.45	64.96±9.48	70.00±13.10	69.65±11.84)
Statistics	*F* = 0.73, *p* = 0.48	*F* = 0.57, *p* = 0.57	*F* = 1.28, *p* = 0.28	*F* = 1.03, *p* = 0.36	*F* = 3.01, *p* = 0.05	*F* = 1.69, *p* = 0.19
**Family type**						
Monogamy	4.68±0.70	3.84±1.04	57.25±8.18	65.62±9.02	72.66±12.02	70.81±9.83)
Polygamy	3.99±0.82	3.87±0.97	55.47±6.06	65.90±7.54	69.56±10.83	71.36±10.05)
Statistic	*t* = 0.79, *p* = 0.43	*t* = 1.49; *p* = 0.14	*t* = 2.42; *p* = **0.02**	*t* = 0.21, *p* = 0.83	*t* = 1.74, *p* = 0.08	*t* = 0.36, *p* = 0.72
**Social support**						
Low	3.84±0.84	3.56±1.04	56.36±8.50	64.00±9.83	72.14±11.81	71.39±12.06)
Average/high	4.01±0.80	3.87±0.07	57.19±7.98	65.85±8.76	72.48±11.97	70.82±9.53)
Statistics	*t* = 0.16, *p* = 0.87	*t* = 0.16, *p* = 0.87	*t* = 0.16, *p* = 0.87	*t* = 0.16, *p* = 0.87	*t* = 0.16, *p* = 0.87	*t* = 0.16, *p* = 0.87
**Gender-based violence**						
Yes	3.9±10.82	3.83±0.98	56.4±38.95	65.58±8.87	72.32±11.77	70.23±11.86
No	4.00±0.81	3.97±0.99	57.14±7.98	66.67±9.43	73.57±14.50	72.2311.85
Statistics	*t* = 0.61, *p* = 0.54	*t* = 0.82, *p* = 0.41	*t* = 0.51, *p* = 0.61	*t* = 0.70, *p* = 0.48	*t* = 0.60, *p* = 0.55	*t* = 0.84, *p* = 0.40
**Childhood experience**						
Pleasant	4.02±0.78	3.87±0.97	57.16±7.91	65.69±8.95	72.66±11.37	71.04±9.62
Unpleasant	3.64±1.11	3.39±1.09	56.06±9.84	64.90±8.14	68.18±18.69	68.37±12.78
Statistics	*t* = 1.936, *p* = 0.06	*t* = 2.69, *p* = **0.01**	*t* = 0.76, *p* = 0.45	*t* = 0.50, *p* = 0.62	*t* = 1.36, *p* = 0.18	*t* = 0.16, *p* = 0.87

s.d., standard deviation; QoL-PHY, quality of life-physical domain; QoL-PSY, quality of life-psychological domain; QoL-SOC, quality of life-social domain; QoL-ENV, quality of life-environmental domain; QoL-QV, quality of life-overall domain; QoL-GH, quality of life-general health domain.

**TABLE 8:** Relationship between quality of life and obstetric/gynaecological characteristics (both trimester).

Variables	QOL-QV Mean (s.d.)	QOL-GH Mean (s.d.)	QOL-PHY Mean (s.d.)	QOL-PSY Mean (s.d.)	QOL-SOC Mean (s.d.)	QOL-ENV Mean (s.d.)
**Planned pregnancy**						
Yes	4.01±0.79	3.86±0.98	57.35±8.48	65.93±8.79	72.50±12.04	71.01±9.57
No	3.85±0.97	3.64±1.02	57.07±7.99	62.81±9.49	71.38±11.03	69.63±12.19
Statistic	*t* = 1.17, *p* = 0.25	*t* = 1.53, *p* = 0.13	*t* = 0.24, *p* = 0.81	*t* = 2.44, *p* = **0.02**	*t* = 0.65, *p* = 0.52	*t* = 0.80, *p* = 0.43
**Parity**						
1st pregnancy	4.01±0.84	4.01±0.93	57.25±8.19	65.86±8.87	72.54±11.88	70.83±10.31
2nd/>	3.97±0.76	3.72±1.01	56.86±7.81	65.50±8.93	72.19±12.06	70.92±9.53
Statistic	*t* = 0.63, *p* = 0.53	*t* = 3.49, *p* = **0.00**	*t* = 0.57, *p* = 0.57	*t* = 0.48, *p* = 0.63	*t* = 0.35, *p* = 0.73	*t* = 0.10, *p* = 0.92
**Abortion**						
Yes	3.66±0.95	3.83±0.99	56.77±8.27	63.15±9.52	65.37±15.98	64.22±13.95
No	4.03±0.78	3.84±0.98	57.13±8.01	65.93±8.79	73.19±11.14	71.64±8.97
Statistic	*t* = 2.93, *p* = **0.01**	*t* = 0.09, *p* = 0.93	*t* = 0.32, *p* = 0.75	*t* = 2.26, *p* = **0.02**	*t* = 3.63, *p* = **0.00**	*t* = 3.96, *p* = **0.00**
**Mode of delivery**						
Normal	4.01±0.85	4.03±0.91	57.26±8.19	65.77±8.84	72.82±12.18	71.17±9.91
C/S	3.98±0.73	3.72±1.01	56.83±7.79	65.44±9.00	71.71±11.54	70.43±10.51
Statistic	*t* = 0.43, *p* = 0.67	*t* = 3.74, *p* = **0.00**	*t* = 0.62, *p* = **0.67**	*t* = 0.42, *p* = 0.42	*t* = 1.08, *p* = 0.28	*t* = 0.88, *p* = 0.38

s.d., standard deviation; QoL-PHY, quality of life-physical domain; QoL-PSY, quality of life-psychological domain; QoL-SOC, quality of life-social domain; QoL-ENV, quality of life-environmental domain; QoL-QV, quality of life-overall domain; QoL-GH, quality of life-general health domain.

**TABLE 9:** Relationship between quality of life sociodemographic and depression in pregnancy.

Variables	QOL-QV Mean (s.d.)	QOL-GH Mean (s.d.)	QOL-PHY Mean (s.d.)	QOL-PSY Mean (s.d.)	QOL-SOC Mean (s.d.)	QOL-ENV Mean (s.d.)
**Both trimester depression**						
Yes	3.68±0.96	3.44±1.18	54.62±10.87	61.08±11.24	66.87±19.35	61.43±14.04
No	4.02±0.79	3.89±0.96	57.29±7.75	66.00±8.60	72.83±11.01	71.62±9.05
Statistic	*t* = 2.18, *p* = **0.03**	*t* = 2.27, *p* = **0.03**	*t* = 1.54, *p* = 0.13	*t* = 2.74, *p* = **0.01**	*t* = 1.90, *p* = 0.06	*t* = 4.57, *p* ≤ **0.01**
**First depression**						
Yes	3.60±1.04	3.17±1.23	54.64±9.43	62.92±11.02	63.61±20.70	60.10±14.21
No	4.09±0.91	3.47±0.98	56.31±0.98	66.44±8.31	66.44±8.31	70.53±9.18
Statistic	*t* = 2.76, *p* = **0.01**	*t* = 1.54, *p* = 0.13	*t* = 1.15, *p* = 0.25	*t* = 2.11, *p* = **0.04**	*t* = 2.11, *p* = **0.04**	*t* = 3.93, *p* = **0.00**
**Third trimester depression**						
Yes	4.02±0.81	4.18±0.60	54.55±4.65	56.06±10.76	73.14±10.82	65.06±13.54
No	3.99±0.81	4.24±0.77	58.20±8.10	65.59±8.85	75.76±14.65	72.63±8.82
Statistic	*t* = 0.35, *p* = 0.72	*t* = 0.27, *p* = 0.79	*t* = 0.82, *p* = 0.43	*t* = 3.47, *p* = **0.00**	*t* = 0.78, *p* = 0.43	*t* = 1.84, *p* = 0.09

s.d., standard deviation; QoL-PHY, quality of life-physical domain; QoL-PSY, quality of life-psychological domain; QoL-SOC, quality of life-social domain; QoL-ENV, quality of life-environmental domain; QoL-QV, quality of life-overall domain; QoL-GH, quality of life-general health domain.

## Discussion

The mean ages reported in two previous studies in Nigeria^12,18^ are similar to that obtained in our study (27.26). In the same vein, similar values (27.29 and 28.8 years) were reported in studies carried out in Nigeria.^2,10,19^ This was so because the mean age fell within the peak reproductive age for women (25–35 years) and thus explained the reason for the similarities observed. By age 40 years, a woman’s chance of getting pregnant drops to less than 10% per menstrual cycle.^20^

About three-quarters of the respondents had at least a secondary education, which is higher than the rate of 30% reported for the general population of women in Nigeria.^21^ This finding may be a consequence of the new social drift where females are encouraged to acquire tertiary education as soon as possible and then subsequently marry to avoid the gynaecological complication associated with ages greater than 34 years. Moreover, the Yoruba ethnic groups from previous studies have been purported to have a higher literacy level with a cultural bias or penchant for education.^22^ In the mane, the predominance of the Yoruba ethnic group in our study is in keeping with other studies done in the southwest of Nigeria that is essentially Yorubas.^12,18^

Our study found that the majority (87%) of the pregnant women were married. This is similar (93%) to another study done in the same locality as our study.^4^ Moreover, Abasiubong^23^ in Nigeria, Choi and colleagues^24^ and Hermann and colleague^25^ both in South Africa reported similar findings. However, a study carried out among pregnant Latin American women^26^ showed about one-third of pregnant women were married. This difference may stem from differences in cultural beliefs about marriage and societal norms among the different races one in the African context and the other a more secular population where being single appears to be trending. In the African culture and indeed in the typical Nigerian society, single parentage is usually frowns at.

The majority of respondents had a secondary education, which is in agreement with a previous study done among Yoruba and Benin women in Nigeria.^12,27^ This may be related to the cultural value attached to education in these ethnic groups where parents regard children attending school as self-actualisation.^22^

The majority of the pregnant women were employed, which was similar to two previous studies from the same setting.^4,28^ However, a study in sokoto^29^ Northern Nigeria reported less than a quarter of pregnant women as employed. This finding may be related to religious, socio-economic and educational differences among the women in the north, as most of these women are known to be poorly educated and are usually full housewives. The predominant family type in our study was monogamy and was in keeping with what was obtainable by Thompson and colleagues in a similar study carried out in this area.^4^ This may be because Abeokuta is majorly a Christian state and Christianity frowns against polygamy as it believes that the Christian doctrine supports the ideas of marriage being between two opposite sexes.

The finding in ourstudy that most of the women reported average to high social support is also in keeping with the study carried out by Thompson et al. However, it differs from a study carried out in Ethiopia^30^ where a higher prevalence of low social support was reported. This may be because Nigerian society supports and encourage extended family bonding. Antenatal clinic attendance was higher among women who have delivered at least once in their lifetime compared with those presenting with their first pregnancy; this is similar to findings of a study among pregnant women in a similar environment.^28^ Furthermore, Williams and colleague^21^ in South Africa also reported similar findings in their study. Women who have delivered once or more may certainly be better informed about pregnancy than nulliparous women and may present more for antenatal care.

In our study, majority of the pregnant women had a planned pregnancy, which is in agreement with that of Thomson and colleague.^4^ Our study was done among married Africans, who believe that childbearing in marriage is essential. The pattern of distribution of socio-demographic and obstetrics and gynaecological variables across trimesters were majorly similar, and there was no significant difference across trimesters except for marital status, occupation status, childhood experience, parity and mode of delivery.

Overall, the prevalence of depression among pregnant women was 7.2%, which was lower than previous studies carried in Nigeria,^4^ Ethiopia,^30^ Omani^31^ and Malawi.^32^ It is worthy of note that the study instrument differed from the current study. However, the findings in our study were similar to two previous studies (9.18% and 8.3%) done in south-west Nigeria^12^ and in a South African population.^33^ When taken together, our study finding is comparable to what obtains in the Nigerian general population^34^ but lower than reported rates in other parts of the world. Healthcare service in Nigeria remains out of pocket, which might make it difficult for people in low- and middle-income people to access care that may not be the case among high-income nations. Thus, a couple of participants might have been invariably exempted, hence the lower prevalence in this climate. Also, the difference could be related to the difference in the methodology employed in the various studies. Nevertheless, the prevalence of 7.2% implies that depression is a common psychiatric morbidity in pregnancy and cannot be overemphasised.

The prevalence of a depressive disorder in the first and third trimesters of pregnancy was 10.5% and 3.9%, respectively. The finding in each trimester is higher than that found in the Nigerian survey of mental health by Gureje and colleagues for depressive disorder.^35^ The finding of lower prevalence of a depressive disorder in the third trimester is also in keeping with that reported by Felice and colleague,^36^ where a lower prevalence of depressive disorder was found in the late pregnancy period compared with the earlier period of pregnancy. This pattern of distribution is, however, different from the study by Abiodun and colleagues^2^ who found no difference in the prevalence of psychiatric morbidity across trimesters. Although his study did not focus on specific psychiatric morbidity but noted that depressive disorder and anxiety disorder were the commonest psychiatric morbidity in pregnancy. An explanation for the high prevalence of a depressive disorder in the first trimester may be that depressive symptoms had started long before pregnancy and was only exacerbated by pregnancy state. Also, the initially increased prevalence of depressive disorder observed in the first trimester may be resulting from the anticipation of getting pregnant.

The high prevalence of depressive disorders in the first trimester suggests that physicians should pay more attention to pregnant women when presenting in their first trimester of pregnancy so that they can identify and manage their emotional challenges and improve the foetal and maternal outcomes. There is also a need for more research on this issue particularly first trimester as a high amount of studies has been done in late pregnancy, and there is a need to establish the variation of psychiatric morbidity across trimesters. The most frequent depressive symptom was psychomotor retardation, which is believed to be a central feature of depressive illness and predicts the severity of depression.^37^ The frequency of anhedonia reported in our study is lower than what was reported in a study carried out in Brazil.^38^

Also, our study revealed that depression in pregnancy was associated with younger age, single marital status and history of miscarriage. The history of previous miscarriage, a negative life event and the fear of a repeat especially when the pregnant woman is getting to accept the pregnancy may precipitate depressive symptoms. The lack of a partner to provide comfort and steady support may explain why depressive disorder was seen more among single pregnant women. This is supported by other researchers who found similar associations.^12,34,39,40,41^ Our study showed that in the first trimester of pregnancy, parity, previous history of miscarriage and history of caesarean section predict psychiatric morbidity in the first trimester. This may be explained by the fact that these are past negative life events and the fear of a repeat especially when the pregnant woman is getting to accept the pregnancy may precipitate depressive illness. Having more than two children especially with the associated financial burden may be a source of the constant worry associated with negative thoughts and thus precipitating depressive symptoms.

Our study found that the previous experience of one or more miscarriages was the only socio-demographic and obstetrics/gynaecological characteristics associated with depression among the respondents and was the independent predictors of depression. This is supported by previous studies where similar associations were made,^9,42,43,44^ one even linking it to increased risk of suicide. However, this association was not found in other studies where the relationship between abortion and depressive illness was evaluated.^45,46^ Our study revealed a significant relationship between being single and having a poor QoL (in the general health domain and the physical domain of QoL). A study carried out in Korea revealed that single women had a worse score than married women.^47^ Likewise, there was a significant relationship between abortion/miscarriage and poor QoL in the overall, psychological and social domain of QoL. This was in keeping with two previous studies in Iran^48^ and Uganda.^49^ It was opined that this relationship was dependent on the level of social support these women received during this period. Women who had a supportive partner reported better psychological support thus influencing their QoL. The finding of poor QoL in the psychological domain in women who have experienced abortion/miscarriage may be due to the grief associated with the event and the sense of loss associated with it.^48^

Having an unpleasant childhood experience was a significant predictor of poor QoL. The severity of childhood maltreatment was significantly related to the severity of QoL during pregnancy in a previous study,^50^ and emotional/psychological trauma and physical trauma in childhood produce significant post-traumatic stress syndrome in pregnancy. A history of previous caesarean section was found to have a significant association with poor QoL in the general health of the QoL domain. This is in keeping with three previous studies conducted among pregnant women in the middle east,^51^, Europe^52^ and America^53^ where it was observed that having a vaginal delivery was significantly better than C/S in terms of physical, mental and emotional/psychological QoL. This is believed to be related to postoperative complications. Meanwhile, a study by Schindl and colleagues^54^ found no difference in QoL between women who delivered vaginally and those who delivered by elective C/S.

Having poor social support also demonstrated a significant association with a poor QoL in the general health domain and psychological domain of QoL. In a study conducted in Japan^55^ among pregnant women, social support during pregnancy was considered a necessary factor in the health and well-being of mothers. It concluded that women with ample social support had fewer complications during pregnancy. It also noted that stress in pregnant women increases with decreasing social support. Older age also showed a significant association to poor QoL in the physical domain and psychological domain of QoL. In a study among menopausal women in South Korea,^56^ women who were younger at their first delivery and who had more deliveries were noted to be at increased risk of health-related QoL problems after menopause. Furthermore, having an unplanned pregnancy also revealed a significant relationship with poor QoL in the psychological domain of QoL. Among pregnant women in Iran,^57^ it was reported that having unplanned pregnancy results in poor QoL with a negative impact on mental health. This was also replicated in a UK study^58^ where pregnant women with unintended pregnancy experienced more psychological distress compared with pregnant women who had planned their pregnancies.

In the first trimester of pregnancy, there was a significant association between being single and having a poor QoL in the overall QoL and environmental domains of QoL. Likewise, there was a significant association between abortion/miscarriage and poor QoL in the overall QoL, psychological domain, the social and environmental domains of QoL. Having an unpleasant childhood experience was found to have a significant association with poor QoL in the overall QoL. Also, having poor social support demonstrated a significant association with a poor QoL in the general health domain and psychological domain of QoL. Older age also showed a significant association with poor QoL in the physical, psychological, social and environmental domains of QoL. Also. having a lower level of education was significantly associated with poor QoL in the physical domain of QoL. Likewise, having a history of a previous caesarean section was significantly associated with having a poor QoL in the environmental domain of QoL.

In the mane, the third trimester of pregnancy was significantly associated with being single and having a poor QoL in the general health domain, psychological domain and environmental domain of QoL. More so, there was a significant association between older age and poor QoL in the psychological domain and social domain of QoL. Likewise, having two or more children and a history of a previous caesarean section was significantly associated with having a poor QoL in the physical domain of QoL.

Among all respondents, there was a significant association between depression and poor QoL particularly in the overall QoL, general health, psychological domain and environmental domain of QoL. In the first trimester of pregnancy, the association between depression and QoL was found to be significant in the overall QoL, psychological domain, social domain and environmental domain of QoL. Among the respondents from the third trimester, depression was only found to be significantly associated with the psychological domain of QoL. These findings of lower QoL in individuals with depression have been replicated in previous studies.^59,60,61^ The relationship between QoL and depression may be bidirectional. Poor QoL may sometimes be a consequence of depression.^62^ In contrast, poor QoL may be a precursor to depression.^63^ Hays and colleagues noted that the effect of depression on QoL was worse than that of chronic medical diseases.^64^ Da Silva Lima and colleagues observed that quality of life may even be impaired in subsyndromal depression.^65^ In the first trimester of pregnancy, the association between depression and QoL was found to be significant in the overall QoL, psychological domain, social domain and environmental domain of QoL. Among the respondents from the third trimester, depression was only found to be significantly associated with the psychological domain of QoL.

Respondents with depressive illness had significantly lower scores on the overall QoL, general health,psychological and environmental domains, which is similar to what was reported in previous studies where a significant negative correlation was found between depression and perceived quality of life,^66,67^ suggesting that respondents with depression reported less satisfaction with their quality of life and vice versa. Beck’s negative cognitive schema of depression could also explain this association as patients with depression are likely to have a negative and irrational view of themselves, their future and the world around them.^68^ There is also an overlap between depression and quality of life as studies have shown that depression and quality of life can predict each other with no clear causal direction and the fact that factors associated with the emergence of depression are also predictors of quality of life.^69^

One limitation of this was the cross-sectional nature of our study, which made it impossible to examine the direction of causality between depressive disorder and their associated socio-demographic and obstetrics/gynaecological characteristics. Our study was carried out in one centre, which may not represent the pattern of depression and QoL among all pregnant women in the country. Future study can improve on this limitation by carrying out the study in multiple centres across the nation. Our study did not screen for anxiety symptoms in respondents. Anxiety is related to pregnancy and could have been exacerbated in pregnancy and could have contributed to the occurrence of depressive illness in pregnancy and affect the perception of an individual’s quality of life in pregnancy. Despite the limitations of the study, to the author’s best knowledge, our study is one of the first attempts to explore significant correlates of quality of life among pregnant women in our environment. It also adds to the emerging body of work on the quality of life of pregnant women in Nigeria. The use of a structured diagnostic instrument on all the respondents allowed the diagnosis of clinical depression and not just mere symptom load. Our study is one of few studies done in Nigeria that has attempted to put into consideration, the relative rarity of first-trimester antenatal clinic attendees and has made effort to adequately represent them in the total study sample. The fact that the current study had based its comparison on trimesters of pregnancy rather than an early or late period of pregnancy makes it to be more specific and contextual.

The results show that there is a high prevalence of depression in pregnant women, and a previous miscarriage is an independent predictor of depression among pregnant women. Thus, there is a need to pay attention to the psychological well-being of pregnant women by routinely screening for depression among them and those identified should be referred to receive appropriate intervention. Also, depression in pregnancy is related to the poor quality of life especially in the social relationship and psychological domains. A conscious effort must therefore be taken to better prepare women generally for pregnancy. Our study also highlights the need to pay closer attention to the psychological well-being and quality of life of all pregnant women and not just on their physical health and the baby’s well-being. Using the background knowledge of possible predictors of depression and poor quality of life such as those highlighted in our study, clinicians should ensure routine screening of the caregivers for depression and institute appropriate intervention. This will help alleviate the severity of depression and improve their quality of life especially in pregnancy that is already a stressful period. Our study emphasises the need for guidelines that will enhance this. The government has a major role to play in improving the quality of life of pregnant women by ensuring a sustainable national economy, promoting education and subsidising antenatal care. Mental health awareness and education are also recommended for the physicians attending to these pregnant women in the clinics, so they are better able to pick up on symptoms of mental and psychological illness. Moreover, the public should also be educated on mental disorders by encouraging culturally acceptable mental health promotion interventions, and this will facilitate a strong social support system for people with mental illness and their family members.

Finally, considering the high prevalence of these disorders among pregnant women, it is important to develop a screening assessment tool that targets the antenatal period. It is necessary to inculcate psychiatric educational programme in the antenatal clinic health talk to serve as a preventive means and create awareness of such among pregnant women. Regular training should be given to health workers in the antenatal clinic to develop a high suspicion of emotional disorders among pregnant women with early referral to mental health liaison service within the secondary care system.
